# BRAZILIAN GASTROENTEROLOGY FEDERATION (FBG) CLINICAL GUIDELINE: DIAGNOSIS AND TREATMENT OF EOSINOPHILIC ESOPHAGITIS IN ADULTS AND ADOLESCENTS

**DOI:** 10.1590/S0004-2803.24612025-079

**Published:** 2025-10-27

**Authors:** Gerson DOMINGUES, Joaquim Prado Pinto de MORAES-FILHO, Décio CHINZON, Áureo DELGADO, FBG advisors

**Affiliations:** 1 Universidade do Estado do Rio de Janeiro, Rio de Janeiro, RJ, Brasil.; 2 Faculdade de Medicina da Universidade de São Paulo, São Paulo, SP, Brasil.; 3 Universidade Federal de Juiz de Fora, MG, Brasil.

**Keywords:** Eosinophilic esophagitis, diagnosis, treatment, complications, Esofagite eosinofílica, diagnóstico, tratamento, complicações

## Abstract

**Background:**

Eosinophilic esophagitis (EoE) is a chronic immune-mediated inflammatory disease of the esophagus that affects both children and adults.

**Objective:**

The purpose of this article is to establish guidance and updates for the diagnosis and treatment of EoE.

**Methods:**

The Brazilian Federation of Gastroenterology, FBG, gathered a group of gastroenterology experts in the field of esophagus and conducted a Delphi process to develop updated guideline for the management of patients with EoE in the light of recent evidence. Grading of the strength and quality of evidence of the recommendations was performed using GRADE criteria. The database searched included MEDLINE, EMBASE, SciELO, LILACS, ClinicalTrials.gov, and CINAHL, using their respective search mechanisms with studies retrieved up to January 2025. The resulting document is presented in the form of 15 questions and answers on diagnosis and treatment, with practical approaches and particularities of EoE, based on the current best evidence-based medicine, indicating the quality of data and specialists’ opinion on the subject.

**Results:**

From the standpoint of diagnosis, the present clinical guideline focused on the most prevalent aspects of EoE as well aiming to promote the benefits of the early diagnosis in clinical practice. The treatment is herein focused on the major advances in pharmacologic approach and therapeutic options available, as well as in the consistent corpus of evidence on dietary therapy.

**Conclusion:**

This FBG guideline is established to support clinical practice and suggest preferable approaches to a typical patient with EoE based on the currently available published literature. In this context, physicians must take into account patient´s medical comorbidities, adherence to treatment and preferences.

## HIGHLIGHTS

• The purpose of this guideline is to provide recommendations on the diagnosis and treatment of EoE based on the available current evidence.

• The diagnosis must meet all three domains of the disease: clinical, endoscopic and histologic findings.

• Options to treatment are elimination diets, swalled topical corticosteroids, proton pump inhibitors and biological therapy as dupilumab. Emphasis should be given to the maintenance treatment for all patients.

## RESUMO

**Contexto:**

Esofagite eosinofílica (EoE) é uma doença do esôfago inflamatória crônica imuno-mediada que acomete crianças e adultos.

**Objetivo:**

O propósito desse artigo é estabelecer orientações e atualizações para o diagnóstico e tratamento da EoE.

**Métodos:**

a Federação Brasielira de Gastroenterologia, FBG, reuniu um grupo de gastroenterologistas com expertise na área de doenças esofágicas e utilizou a metodologia Delphi para desenvolver uma diretriz atualizada de conduta para pacientes com EoE à luz da medicina baseada em evidências. A graduação da força e qualidade de evidência das recomendações foi realizada usando os critérios do Sistema GRADE. A pesquisa dos dados incluiu MEDLINE, EMBASE, SciELO, LILACS, ClinicalTrials.gov e CINAHL, utilizando seus respectivos mecanismos de busca com artigos coletados até janeiro de 2025. Esse documento está apresentado na forma de 15 perguntas e respostas sobre o diagnóstico e tratamento da EoE que abordam questões práticas, particularidades da EoE com base nas melhores evidências para cada assunto, sinalizando a qualidade da informação, quando possível e na opinião de especialistas.

**Resultados:**

Do ponto de vista do diagnóstico, a presente diretriz clínica focou nos aspectos mais prevalentes da EoE, assim como, nos benefícios do diagnóstico precoce da doença na prática clínica.No tratamento foram observados os avanços mais recentes da terapia farmacológica e as opções de tratamento disponíveis, como também, no corpo robusto de evidências disponíveis para a terapia dietética.

**Conclusão:**

Essa diretriz da FBG estabelece um suporte para a prática clínica e sugere condutas preferenciais para os pacientes com EoE baseadas na literatura atual. Nesse contexto, os médicos devem considerar outras comorbidades associadas dos pacientes, aderência ao tratamento e preferências.

## INTRODUCTION

Eosinophilic esophagitis (EoE) is a chronic esophageal condition that has been identified and studied in the last three decades. It is characterized by esophageal dysfunction with clinical outcomes and impairments of high impact to the patient. EoE incidence and prevalence increased substantially and heterogeneously in regions around the world^1^, partially by clinical features of the disease that may be confounded with other conditions. Thus, it is timely to emphasize the requiring uniqueness of the ensemble issues as it affects hundreds of thousands of patients with a steadily growing burden of care for patients and healthcare systems^2^ ^,^ ^3^.

The crescent corpus of data and evidence in research articles are mainly focused in EoE pathophysiology, diagnostic criteria, disease management and guidelines. Nonetheless, a Brazilian guideline was not created so far and is paramount for a population with an acknowledgedly heterogenous genetic profile. Such scenario urges for gathering evidence to the practician. Thus, the purpose of the present article is to produce an up-to-date guideline under the curation of a recognized corpus of specialists of the Brazilian Gastroenterology Federation (Federação Brasileira de Gastroenterologia - FBG) to fill a gap in the literature. The resulting document presents practical approaches, particularities of and differential diagnostic, finally presenting the treatment of EoE based on the current best evidence-based medicine, indicating the quality of data, risk of bias and specialists’ opinion on the subject.

The condition is defined as a type 2 inflammation characterized by overexpression of type 2 cytokines, causing elevated activity of mediators and inflammatory cells, admittedly as the drivers of the histopathological features commonly seen in EoE after a trigger stimulus^4^. Under this definition, EoE is a chronic allergic disorder characterized by an infiltrate in the esophageal mucosa predominantly eosinophilic, epithelial thickening and fibrosis, resulting in esophageal dysfunction, i.e, dysphagia, majorly in adolescents and adults^5^. Currently EoE is considered the main cause of food impactions in the emergency endoscopic suite^6^. The slow progression of the disease, incompletely or untreated disease, as well as gaps during the treatment are the culprit of the ever-growing severity of the EoE as it evolves from a manageable inflammatory condition to an irreversible fibrostenotic phenotype in most of the patients^5^ ^,^ ^7^ ^,^ ^8^.

From the standpoint of diagnosis, this clinical guideline focused on the most prevalent aspects of the primary stages of the condition aiming to promote the benefits of the early diagnosis in clinical practice.

The treatment is herein focused on the major advances in pharmacologic treatment and therapeutic options available, as well as in the consistent corpus of evidence on dietary therapy^9^. Moreover, the approach adopted in this guideline was to highlight the clinical practice and evidence-based recommendations encompassed with the evolution of EoE diagnosis and treatment, also considering many recently published consensus statements from other countries^5^ ^,^ ^9^ ^-^ ^11^. Nonetheless, the scarcity of top level and high-quality evidence prescribes discretion and caution of the practitioner in the clinical setting. In order to tackle the gaps and many other issues faced in the construction of this guideline, we seek for expert opinion and aiming to provide possible approaches. Thus, it is highly recommended to consider the information regarding the quality of the evidence in every section.

Finally, this guideline is intended to provide support to the clinical practice and the approaches to EoE herein presented are based on the better evidence available to enhance clinical decision-making and counseling^3^ ^,^ ^7^ ^,^ ^9^ aligned with the consensus of the authors and the FBG in the cases of absence data, conflicting or lack of evidence. The development of an updated protocol was driven by several factors, such as the introduction of new diagnostic tools (requiring reassessment of diagnostic criteria and practices) and the adoption of therapeutic alternatives for the condition, leading to certain changes in current clinical practice^11^ ^,^ ^12^ ^,^ ^13^.

Thus, this guideline was based in the typical EoE patient and the most prevalent features according to the definitions and mechanisms currently available. Needless to point the unsurmountable relevance and the impact of comorbidities, adherence to treatment, patients’ needs and preferences with which physicians may find to properly provide and manage the condition. Table 1 summarizes the current recommendations.

**TABLE 1:** Recommendations for diagnosis and treatment of EoE.

Recommendation	Quality of evidence	Level of agreement
EoE is a Th2 antigen-mediated disease characterized by esophageal dysfunction and the presence of ≥15 eosinophils per high-power field in the esophageal mucosa, in the absence of other causes of esophageal eosinophilia.	High	100%
Dysphagia and food impaction are the most frequent symptoms, although manifestations similar to GERD may also occur.	Intermediate	100%
The endoscopic features of EoE include edema, exudates, longitudinal furrows, rings, strictures, narrowing, and crepe-paper mucosa.	High	100%
Histological findings in EoE are the presence of eosinophils (≥15 eosinophils per high-power field), basal layer hyperplasia, dilated intercellular spaces, mast cells and lymphocytes, eosinophilic degranulation, eosinophilic microabscesses, and subepithelial fibrosis.	Intermediate	100%
Esophageal mucosal biopsies should be obtained from the upper, middle, and lower thirds of the esophagus, with at least six specimens, even if the endoscopic appearance is macroscopically normal.	High	92%
Allergy testing has very limited applicability.	High	100%
Management of the disease		
Empirical elimination diets are the most effective therapeutic option.	High	100%
Proton pump inhibitors (PPIs) are effective in the treatment of EoE, with no difference in response between different PPIs.	Intermediate/High	100%
Swallowed topical corticosteroids are effective in treating EoE, with no significant differences among available formulations.	Intermediate/High	100%
The biologic agent dupilumab is effective for EoE treatment at all stages of the disease.	Intermediate/High	100%
Maintenance pharmacologic treatment is recommended for all patients with EoE.	High	100%
Complications of EoE are infrequent and include food impaction, esophageal perforation, and esophageal motility disorders.	Intermediate	92%
Endoscopic dilation is effective in improving symptoms in patients with fibrostenotic disease. A luminal diameter of 16 mm is considered a satisfactory endpoint.	High	92%
Patients with EoE should be followed with regular clinical consultations and endoscopic assessments, even if asymptomatic.	Intermediate	100%
Early diagnosis and treatment reduce the risk of complications associated with EoE and improve quality of life.	Intermediate/low	100%

## METHODS

The development of this guideline followed the steps outlined below

Thematic review of the literature to develop the PICO question (from the acronym patient, intervention, comparison, outcomes), guiding the organization, evaluation and validation of the questions, which are the structural axis of this document.

Conduct of an integrative literature review addressing EoE for each initially defined question.

Assessment of the quality of evidence based on the GRADE protocol (grading of recommendations assessment, development, and evaluation)^14^.

Submission of the review to a panel of specialists who, through the delphi convergence approach, defined the level of agreement regarding the evidence presented.

Following the previous step, the text was revised to incorporate identified modifications, producing the final structure of the work.

### Development of guiding questions

Members of the Scientific Events Department of the Brazilian Gastroenterology Federation (FBG) drafted initial questions based on their clinical experience, summarizing the main pertinent and necessary information for the EoE guideline. These questions were reviewed by a working group of specialists in the field, resulting in a set of items framed as questions to be answered.

### Integrative review

This type of investigation seeks to aggregate evidence to guide clinical practice effectively^15^ and reduce individual preferences in the implementation of evidence^16^. Its strength lies in how evidence from clinical trials acquires the status of practical knowledge, rather than purely scientific rigor, fostering practices based on published articles, often without considering the context in which the trial data were generated and how they should be appropriately used.

The variables to be studied^17^ were defined by FBG and presented in the form of questions, each bringing with it the criteria for evidence research. After answering the questions, the selected articles, when applicable, were evaluated for the quality of the evidence.

### Meta-analyses

In order to provide a graphic element for rapid consultation, a systematic review with meta-analysis was conducted, focusing on selected items curated by the authors to optimize the clinical decision-making process. This quantitative synthesis was limited to items for which available data permitted such an approach.

The PRISMA statement^14^ was adopted but not applied in its entirety, only when it unequivocally contributed to the quality of the work. For the same reason, no prior registration of the meta-analyses presented herein was performed.

The databases searched included MEDLINE, EMBASE, SciELO, LILACS, ClinicalTrials.gov, and CINAHL, using their respective search mechanisms. No restrictions were imposed regarding language, publication date, study design, or sample size, with studies retrieved up to January 2025. Review articles, case reports, in vitro or animal studies, editorials, letters, opinions, and non-peer-reviewed articles were excluded.

### Eligibility criteria and definition of outcomes

The diagnosis of EoE was based on clinical symptoms of esophageal dysfunction, suggestive endoscopic findings, histologic confirmation of more than 15 eosinophils per high-power field (hpf), and exclusion of other diseases causing esophageal eosinophili^9^. 

### The primary therapeutic outcomes considered were:

Histologic remission, defined as a reduction in the number of eosinophils per hpf to below the reference threshold (<15 eos/hpf)^18^;

Clinical remission, defined by broader criteria related to clinical improvement of patients, as assessed by various instruments, whether externally validated or not^18^.

### Inclusion and exclusion criteria

The common inclusion criteria across all items were: adolescent or adult individuals without comorbidities, presenting clinical, endoscopic, and histologic abnormalities in accordance with the guidelines available up to the time of this document’s preparation^18^.

Exclusion criteria included studies with treatment durations shorter than 12 weeks, use of molecules not authorized or registered in the country, and studies involving pediatric patients.

### Data extraction

Extracted data included: author, year of the study, number of cases meeting the primary outcome, total number of participants, treatment modality, measured outcome (clinical or histologic), treatment duration, medication dosage, and presence of a comparison group.

Data availability or possibility of data extraction were performed regardless of the primary objective of the original study if they provided data of interest to the present guideline.

### Statistical analysis

Meta-analyses of binary outcomes^19^ and single-arm analyses were performed to estimate the proportion of successes in treatments^20^. In both types of meta-analyses, a random-effects model was the primary choice due to anticipated heterogeneity, although a common-effects model was also employed to provide a comparative perspective on the effects^21^.

The same parameters were applied across all meta-analyses to facilitate comparability between interventions. Thus, calculations were conducted using the Mantel-Haenszel method and the Hunter-Schmidt method to estimate between-study variance (τ²) and its square root (τ), with 95% confidence intervals (95%CI)^22^.

Given the expected high variance, Knapp-Hartung adjustments were used to determine the confidence intervals for the summary effect estimates. Heterogeneity was estimated using the I2 statistic^23^, with values below 25% considered low, around 50% considered low to moderate, 50-75% as moderate heterogeneity, and values greater than 75% indicating high heterogeneity.

In all forest plots, studies were ordered according to the magnitude of the calculated effect size. This approach was chosen to provide a more intuitive organization of results, highlighting the range of effect variability across analyses and facilitating the visualization of subgroup differences.

The number of studies and comparisons, as well as the total number of patients, were displayed in each respective item. Statistical analyses were performed using RStudio version 1.1.383 (The R Foundation for statistical computing, Vienna, Austria), utilizing the ‘meta’ and ‘metafor’ packages.

### Assessment of the quality of the evidence

The assessment of evidence quality was performed using the GRADE system^14^ that results in four levels as a whole: high, moderate, low, and very low. Also in five levels for item. This is the most used and recommended system, despite occasional criticisms regarding its objectivity.

Evidence quality was classified from A to E, as defined by the GRADE system (Table 2)^14^, and was further supported using risk of bias assessment tools employed in systematic reviews and meta-analyses. The RoB 2 (Risk of Bias 2) tool was used for meta-analyses of randomized clinical trials, while the Newcastle-Ottawa Scale was applied for meta-analyses of observational studies.

**TABLE 2:** Quality of evidence according to GRADE^14^.

GRADE	Quality of evidence	Qualification criteria
A	High	Data from meta-analyses, systematic reviews, and randomized controlled trials with blinding.
B	Intermediate	Data from observational studies, cohort studies, case-control studies, and randomized trials with limited sampling.
C	Low	Observational data with methodological limitations, moderate to high risk of bias, and retrospective studies.
D	Very low/arguable	Any study design published in non-indexed journals, studies with experimental design limitations, theses, dissertations, editorials, letters, case reports, and expert opinions.
E	Not applicable	Definitions of terms and concepts, information not involving experimental data, and narrative reviews.

Delphi convergence cethod and expert consensus

The adaptation of the Delphi methodology for opinion validation in guidelines or consensus documents has long been used as a means to combine the power of the Delphi method with the objectivity required for the development of a guideline or consensus.

It consists of transforming proposed questions and answers into a structured questionnaire with a five-point Likert-like scale (1=strongly disagree to 5=strongly agree) in electronic form.

The questionnaire developed was sent to the FBG members for their evaluations. Questions with ratings of 4 or 5 below 80% of total responses were subjected to text revision based on respondents’ comments and subsequently re-evaluated. Items unresolved after two voting rounds were rejected.

### Presentation and dissemination of the guideline

The guidelines were designed to be easily incorporated into clinical activity, with practical value to make diagnosis and treatment more assertive. The guidelines will be disseminated through publication and presentation at national and regional meetings.

### What is the concept of EoE?

#### Recommendation

EoE is a Th2 antigen-mediated disease characterized by esophageal dysfunction and the presence of ≥15 eosinophils per high-power field (hpf) in the esophageal mucosa, in the absence of other causes of esophageal eosinophilia.

**Quality of evidence:** High

**Delphi consensus level:** 100%

### Summary of evidence

EoE is a Th2 antigen-mediated disease characterized by esophageal dysfunction (e.g., dysphagia and food impaction) and histological findings demonstrating, via optical microscopy, the presence of ≥15 eosinophils per hpf (or ≥15 eosinophils/0.3 mm² or >60 eosinophils/mm²), in the absence of other causes of esophageal eosinophilia^9^ ^,^ ^10^.

It is an inflammatory condition of the esophagus, currently recognized as a distinct clinicopathologic entity, constituting the second most prevalent cause of chronic esophagopathy after gastroesophageal reflux disease (GERD). It is the leading cause of dysphagia and food impaction in adults and adolescents, and of abnormal feeding behaviors, especially in children^23^, with significant impact on patients’ quality of life^23^.

The eosinophil count in the mucosa (≥15/hpf) is an important diagnostic criterion, established by consensus based on systematic literature review and expert opinion^24^.

All patients with esophageal eosinophilia must be evaluated for non-EoE diseases that may cause or contribute to this condition^18^. Among the diseases we include eosinophilic gastrointestinal disorders, connective tissue diseases, vasculitis, hypereosinophilic syndrome, Crohn’s disease, achalasia, celiac disease, drug hypersensitivity reactions, and - most commonly - GERD^11^. Nevertheless, these conditions are typically distinguishable from EoE by unique clinical features. The diagnostic criteria apply across all age groups^25^.

It is important that the definition of EoE be uniformly adopted among adult and pediatric gastroenterologists, allergists, pathologists, general practitioners, and researchers^26^.

### What is the clinical presentation of EoE?

#### Recommendation

Complaints of dysphagia and food impaction are frequent in EoE, although manifestations similar to those of GERD may occur (heartburn, regurgitation, non-cardiac chest pain). Thus, EoE should be considered as a diagnostic hypothesis in any patient presenting with dysphagia and/or food impaction.

**Quality of evidence:** intermediate

**Delphi consensus level:** 100%

### Summary of evidence

The clinical presentation of EoE includes symptoms of esophageal dysfunction related to inflammation of the esophageal mucosa and/or fibrostenosis in the most advanced stage of the disease. In most cases, the clinical presentation in adults and adolescents is associated with dysphagia (70%) and food impaction (30%)^27^.

The clinical presentation of EoE includes symptoms of esophageal dysfunction related to mucosal inflammation and/or fibrostenosis in advanced disease stages. In most cases, adults and adolescents present with dysphagia (70%) and food impaction (30%)^27^.

Food impaction is particularly notable, as many cases (approximately 50%) require urgent endoscopic removal, leading to the definitive diagnosis of EoE^28^ ^-^ ^30^. Some patients report difficulty swallowing associated with the inability to swallow saliva^31^.

Thus, the most frequent clinical manifestations of EoE include^20^ ^,^ ^32^- ^33^.

- Dysphagia: difficulty swallowing, especially solid foods, typically worsening with disease progression.
- Food impaction: sensation of food blockage in the esophagus, often requiring urgent endoscopic intervention.
- Heartburn and other GERD-related symptoms, such as regurgitation and chest pain, as well as postprandial hypotensive syndrome, characterized by abdominal discomfort.

The clinical presentation varies with disease evolution, age, and ethnicity. Presentation in children may differ from that in adults. Dysphagia and food impaction increase with age and are more common among Caucasians than in other ethnic groups^20^ ^,^ ^21^.

Food impaction requiring endoscopic extraction may be the initial manifestation of EoE. Overall, symptom severity does not strongly correlate with histological severity or eosinophil count in esophageal mucosa^22^ ^,^ ^23^.

Most patients (75%) present at least one associated atopic condition, including food or environmental allergies, atopic dermatitis, allergic rhinitis, nasal polyps, or asthma^30^ ^,^ ^31^.

Symptoms may be present long before diagnosis - sometimes up to 10 years - increasing the risk of developing esophageal fibrosis^20^. Patients may experience seasonal variability in symptoms, with reduced intensity during winter months. EoE diagnosis may be more frequent in summer, underscoring the role of aeroallergens^32^.

### What are the endoscopic findings in EoE?

#### Recommendation

The diagnosis of EoE requires endoscopy with biopsy. A normal-appearing mucosa on endoscopic examination does not exclude the diagnosis of EoE. Endoscopic features of EoE reflect its natural history, comprising active inflammation (edema, exudates, and longitudinal furrows) that may lead to fibrostenotic remodeling of the esophagus (rings, strictures, narrowing, and crepe-paper mucosa).

**Quality of evidence:** high 

**Delphi consensus level:** 100%

### Summary of evidence

Although no pathognomonic findings exist, the principal endoscopic findings characterizing EoE are (Table 3)^34^:

**Table:** 

Signs of Inflammation	Signs of fibrostenosis
Longitudinal furrows/ridges and valleys	Fixed esophageal rings
Exudates/white spots	Trachealization
Pale and edematous mucosa	Feline esophagus appearance
Reduced vascularity	Diffuse or focal esophageal rings
Friable mucosa	Reduced esophageal caliber
Crepe-paper esophagus with lacerations upon endoscope passage	Esophageal stricture

Esophageal rings: concentric mucosal rings resembling trachealization.

Exudates or white plaques: adherent plaques or white spots, sometimes resembling esophageal candidiasis; coalescence forms eosinophilic abscesses.

Esophageal narrowing or strictures: chronic inflammation may result in strictures.

Friable esophageal mucosa: minimal trauma during endoscopy can cause mucosal tears; crepe-paper esophagus presents as delicate, ulcerating mucosa.

Edema and reduced vascularity: edematous mucosa may show reduced vascular markings.

Furrows: longitudinal fissures, distinguishable from erosions seen in GERD, enhanced by air deflation or topical staining.

Edema (pallor due to loss of vascular pattern), exudates (white plaques or spots), and longitudinal furrows are associated with active inflammation, while esophageal rings (trachealization), strictures, narrowing, and crepe-paper esophagus indicate fibrotic remodeling.

**FIGURE 1:**
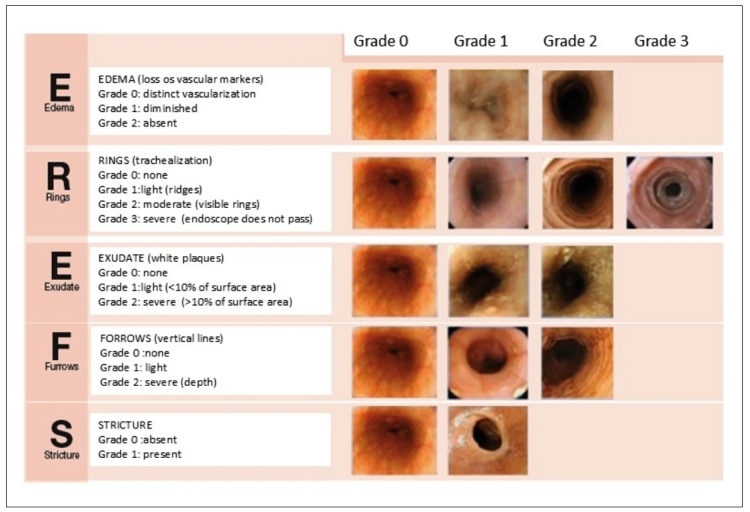
Endoscopic Reference Score (EREFS) classification developed to standardize the diagnosis and severity of EoE. Modified from Hirano et al^36^.

The Endoscopic Reference Score (EREFS) system assigns grades based on characteristic endoscopic findings of EoE, aiming to validate and standardize diagnosis and disease severity^35^. This system evaluates transient or fixed rings, exudates, furrows, mucosal fragility (crepe-paper esophagus), edema, vascular pattern, and strictures (Figure 1)^36^.

Meta-analysis of prospective studies found at least one characteristic finding in more than 90% of EoE patients undergoing upper endoscopy. Sensitivity levels were modest (15-48%), but specificity levels were high (90-95%). Positive predictive values ranged from 51-73%, and negative predictive values from 74-84%^37^.

The EREFS score should be routinely applied to all patients in the diagnosis and also to evaluate treatment response: a reduction in the endoscopic score corresponds with histologic improvement^38^.

### What are the histopathological findings in EoE?

#### Recommendation

The following histologic findings are observed in EoE: presence of eosinophils (≥15 eosinophils per hpf or ≥60 eosinophils/mm²). Other findings include basal zone hyperplasia, eosinophil abscess, eosinophil surface layering, dilated intercellular spaces, surface epithelial alteration, dyskeratotic epithelial cells, lamina propria fibrosis.

**Quality of evidence:** intermediate

**Delphi consensus level:** 100%

### Summary of evidence

Histopathological findings in esophageal tissue vary depending on disease duration, severity, treatment history, and comorbid conditions like GERD. Eosinophilic infiltration is the hallmark finding on esophageal biopsy in EoE.

Hematoxylin and eosin (H&E) staining highlights eosinophils and associated findings like eosinophilic microabscesses^39^. Thus, epithelial eosinophilia, in conjunction with clinical symptoms of esophageal dysfunction, serves as a diagnostic criterion^26^.

While various histological thresholds have been proposed, international guidelines endorse the cutoff of ≥15 eosinophils per hpf^18^ ^,^ ^19^ ^,^ ^26^, with subsequent data supporting its sensitivity and specificity^40^. Although randomized trials to determine the optimal eosinophilic cutoff are lacking, prospective studies demonstrate excellent diagnostic performance for the 15 eos/hpf threshold, with sensitivity of 100% and specificity of 96%^41^.

Eosinophil counts are also used to assess treatment response and monitor disease activity^40^, ideally aiming for eosinophil elimination, although some trials consider <15 eos/hpf acceptable.

The inflammatory response in EoE extends beyond the epithelium with promotion of lamina propria fibrosis. Additional histological features include basal zone hyperplasia, eosinophil abscess, eosinophil surface layering, dilated intercellular spaces, surface epithelial alteration, dyskeratotic epithelial cells increased mast cells and lymphocytes, and papillary elongation^44^.

These features are incorporated into two validated histologic scoring systems: the EoE Histology Scoring System (EoEHSS) and the Eosinophilic Histology Remission Score (EoEHRS), which grade eight histological alterations based on severity and extent on a four-point scale^39^ ^,^ ^42^.

Although developed for clinical trials as these scores have shown high interobserver agreement after basic pathologist training, the recommendation is that the EoEHS should be incorporated into the follow-up of patients undergoing treatment^43^.

### What is the endoscopic biopsy protocol for the diagnosis of EoE?

#### Recommendation

Mucosal biopsies of the esophagus should be obtained from the upper-middle, and distal parts, preferably targeting areas with the greatest abnormality, with at least six biopsy fragments collected, ideally in separate containers, even in patients with macroscopically normal endoscopic findings. During the index endoscopy, biopsies of the stomach and duodenum are recommended to exclude eosinophilic gastrointestinal diseases.

**Quality of evidence:** high

**Delphi consensus level:** 92%

### Summary of evidence

Location: biopsies should be taken from the proximal-middle, and distal esophagus to ensure comprehensive sampling.

Number of biopsies: six to eight biopsies are recommended, focusing on areas of greatest mucosal abnormality to increase diagnostic yield.

Handling and processing: biopsy specimens should be immediately placed in buffered formalin or another appropriate fixative to preserve tissue architecture.

Labeling samples: Each biopsy specimen should be placed in separate containers labeled according to the site of origin (proximal-middle, distal) for correlation with endoscopic findings.

Multiple biopsies are necessary for effective EoE diagnosis due to the patchy distribution of eosinophilic infiltration. At least six fragments from two different sites (usually distal and proximal halves) are recommended to maximize diagnostic sensitivity^39^ ^,^ ^44^ ^,^ ^45^.

Furrows and exudates, which contain intense eosinophilic infiltration, should be targeted preferentially during biopsy. Biopsies should be taken from longitudinal furrows rather than between furrows, where eosinophils are more concentrated. Rings and strictures, which reflect subepithelial fibrosis, are less favorable targets due to weaker correlation with active eosinophilic inflammation^28^ ^,^ ^46^.

Biopsies should also be performed even when the esophageal mucosa appears normal, as eosinophilic infiltration may be present in 10-32% of adult and pediatric patients^19^.

It is advisable to perform duodenal and gastric biopsies at initial diagnosis to rule out eosinophilic gastroenteritis^19^.

### What is the applicability of allergy testing for the diagnosis of EoE?

#### Recommendation

Allergy tests have very limited applicability for the diagnosis of EoE.

**Quality of evidence:** intermediate/high

**Delphi consensus level:** 100%

### Summary of evidence

EoE is characterized as a chronic, immune-allergic esophageal disorder of multifactorial etiology, in which interaction between food and inhalant allergens with the esophageal mucosa in predisposed individuals induces cytokine production, leading to Th2 lymphocyte activation and the release of IL-4, IL-5, IL-13, and TGF-β, without IgE-mediated mechanisms^47^ ^-^ ^49^.

In general, allergy tests detect specific IgE reactions (skin prick tests or serum-specific IgE), specific IgG4, or contact-mediated responses^47^ ^-^ ^49^. Thus, they are not applicable for the diagnosis of EoE.

Similarly, although allergy-test-guided diets were considered a practical approach to identifying EoE food triggers, studies in both children and adults have shown poor concordance between allergy tests and true trigger foods, limiting their applicability^50^ ^-^ ^52^.

According to a meta-analysis by Arias et al.^53^, elemental amino acid-based diets achieve about 90% efficacy in inducing remission, six-food elimination diets achieve 72.1%, whereas allergy-test-guided elimination diets achieve only 45.5%. A systematic review by Pitsios et al47 also evaluated the effectiveness of elimination diets based on allergy tests, showing that they are effective in 66% to 88.3% of cases (combining the results of the detection of specific IgE tests with patch tests), which corroborates previous data that diets directed by allergy tests are not superior to empirical diets^47^.

Thus, allergy testing may be useful to confirm or exclude parallel diagnoses, such as IgE-mediated food allergies or respiratory allergies, but they do not play a significant role in the diagnosis or treatment of EoE.

### What is the applicability of dietary therapy in EoE?

#### Recommendation

Among the three modalities of dietary therapy, empirical elimination of food items has emerged, from a practical medical perspective, as the most effective therapeutic approach, initially favoring the removal of one or two foods (cow’s milk and wheat). However, the choice of diet should be individualized based on medical, nutritional, and patient preference factors. Periodic monitoring of adherence to the proposed diet is essential.

**Quality of evidence:** high

**Delphi consensus level:** 100%

### Summary of evidence

EoE is classified among gastrointestinal diseases with immunologically mediated responses to food antigens^54^. Given that EoE is an allergic disease not mediated by IgE, elimination of allergens through dietary therapy can result in remission in some patients^55^.

### Dietary approaches in EoE can include three main strategies:

Elemental diet;

Empirical elimination diet;

Allergy-test-based elimination diet.

### Elemental diet

The elemental diet consists of a liquid formulation based on essential amino acids, avoiding any proteins. Different studies have considered this possibility, mainly in children, with a meta-analysis summarizing the effectiveness of the elemental diet in inducing histological remission in 90.8% of patients, with clinical and endoscopic improvement^53^ ^,^ ^56^.

A recent systematic review, considering 431 patients, reports histological remission (<15 eosinophils/pg) in 93.6% of patients when compared with 13.3% in the historical placebo group^57^.

The importance of allergens contained in food may, therefore, constitute a contributing factor in EoE, a hypothesis also supported by the recurrence of symptoms after reintroduction of triggering foods and the limited expression of inflammatory cytokines^20^.

However, there are some difficulties in implementing the elemental diet, which is composed of amino acids, fats, sugars, vitamins and nutrients. The unpleasant taste, nutritional concerns, maintenance of body weight and relatively high costs are points to be considered^55^. The elemental diet is a very effective approach (90%), but it seems relatively unfeasible in clinical practice, with patient adherence being one of the main limitations^19^. Newer dietary formulas with improved palatability may increase patient acceptance.

Therefore, although there is evidence of the effectiveness of elemental diets, the recommendation for their use is relatively restricted, especially when considering other dietary and pharmacological therapeutic possibilities^57^. The European Guidelines, for example, consider that elemental diets should only be considered after demonstrating dissatisfaction with pharmacological therapeutic treatment and/or elimination diet, conditions that, in clinical practice, occur more rarely^19^.

The elemental diet consists of a liquid formulation based on essential amino acids, avoiding any proteins. Different studies have considered this possibility, mainly in children, with a meta-analysis summarizing the effectiveness of the elemental diet in inducing histological remission in 90.8% of patients showing clinical and endoscopic improvement^53^ ^,^ ^56^. A recent systematic review consisting of 431 patients reported histological remission (<15 eosinophils/pg) in 93.6% of patients when compared with 13.3% in historically observed placebo group^57^. The importance of allergens contained in food may, therefore, be a contributing factor in EoE, a hypothesis also supported by the recurrence of symptoms after reintroduction of triggering foods and the limited expression of inflammatory cytokines^20^.

However, there are practical difficulties with implementing elemental diets, which consist of amino acids, fats, sugars, vitamins, and nutrients. Unpleasant taste, nutritional concerns, maintenance of body weight, and relatively high costs must be considered. Although highly effective (90%), elemental diets seem relatively impractical in clinical practice, with adherence being a major limitation^19^. Newer formulations with improved palatability may enhance acceptance by patients.

Therefore, despite evidence of efficacy, recommending elemental diets remains relatively restricted, especially considering the availability of other dietary and pharmacological therapeutic options^57^. European guidelines, for example, recommend that elemental diets only be considered after dissatisfaction with pharmacologic therapy and/or empirical elimination diet, conditions that, in clinical practice, are relatively rare^19^.

### Empirical elimination diet

Empirical elimination of food groups commonly related to allergic reactions is a particularly interesting dietary option. It consists fundamentally of excluding foods most frequently associated with EoE, such as milk, wheat, eggs, soy, peanuts, nuts, and seafood (fish and shellfish)^20^ ^,^ ^58^.

A recent systematic review of nine observational groups totaling 633 patients revealed that empirical elimination was associated with histological response in 67.9% of patients (<15 eos/hpf), compared to 13.3% in historical placebo groups^59^.

For the empirical elimination diet, there are options for removing the six foods mentioned above at once (SFED - Six food elimination diet), four foods (FFED - Four food elimination diet), two foods (TFED - Two food elimination diet) and just one food (OFED - One food elimination diet)^59^.

Our meta-analysis revealed that the results for the subgroups indicated that the difference in efficacy between the SFED and FFED diets is small. However, the difference between SFED and TED and/or SPT (skin prick test) can be almost twice as large in favor of SFED. It is worth noting that the heterogeneity (I2) of the subgroups ranged from a minimum of I2=53% for FFED to I2=76% for SFED, considered moderate in all modalities.

These results suggest that overall dietary therapy success rates in adults are around 48%, with a 95% confidence interval of 39-56% in random-effects models and moderate heterogeneity. Nonetheless, chi-square tests showed statistically significant differences among subgroups, although numerical differences were modest due to differences of subgroup sample sizes.

Recent consensus tends to adopt a step-up approach, beginning with elimination of only one or two foods, mainly milk and wheat^59^.

However, limitations to the use of empirical elimination diets are related to practical concerns. The everyday use of an incomplete or inconsistent diet for anti-allergic purposes or poor long-term adherence of patients may limit its applicability impairing the use of empirical elimination diets^59^.

### Allergy-test-guided elimination diet

The recognition that EoE represents a particular form of food allergy, coupled with the difficulties in implementing elemental diets, led to research on identifying food allergy triggers through direct testing.

Allergy testing has been based on single-arm observational studies without comparative composition designs as in the cases of elemental diet and empirical elimination diet. Twelve studies demonstrated that 49.2% of individuals undergoing allergy-test-guided elimination did not achieve histological remission (<15 eos/hpf). The estimated relative risk of failure for histological remission compared to historical placebo was 0.5757,60. Sensitivity analyses revealed no significant differences among studies that adopted correction tests and those that did not^57^.

Therefore, food allergy tests cannot be considered or used as a reference or guidance for dietary management in patients with EoE, as they are insufficiently accurate to justify exclusion of foods as potential disease triggers. Until comparative, placebo-controlled trials with rigorous observational criteria are conducted, allergy-test-guided dietary therapy should not be considered appropriate^56^.

### What is the role of acid secretion inhibitors (Proton Pump Inhibitors [PPIs] and Potassium-Competitive Acid Blockers [P-CABs]) in the treatment of EoE?

#### Recommendation

Treatment with PPIs is effective in achieving clinical and histological remission in a significant proportion of patients with EoE, with no difference in response among the various PPIs. They may be used as first-line pharmacologic therapy, as well as following therapeutic failure of swallowed topical corticosteroids. P-CABs need further studies before establishing their use in clinical practice.

**Quality of evidence:** intermediate/high

**Delphi consensus level:** 100%

### Summary of evidence

The action of PPIs is by inhibiting IL-4-stimulated expression of eotaxin-3 in esophageal cells of patients with EoE, as well as by blocking STAT6 binding to its promoter. Eotaxin-3 is an eosinophil chemoattractant that contributes significantly to the pathophysiology of Th2-mediated esophageal eosinophilia. Overall, about 50% of EoE patients respond to high-dose PPI therapy^61^.

A recent systematic review and meta-analysis including 33 studies and 619 patients under suspected EoE showed that PPIs led to histological remission (defined as <15 eos/hpf) in 50.5% of cases (95%CI 42.2-58.7%) and symptomatic improvement in 60.8% (95%CI 48.38-72.2%). No significant differences were observed regarding patient age, study design, or type of PPI prescribed. The efficacy of PPIs was independent of the presence of pathological acid exposure as shown by esophageal pH monitoring^62^. PPIs are often used as first-line treatment due to their good cost-effectiveness profile and relatively few adverse effects. It is recommended that they be administered for at least 8 to 12 weeks at double doses, before meals, to induce disease remission. Effectiveness should be evaluated by endoscopy with biopsies^63^.

In case of interruption of the pharmacologic treatment, the recurrence of symptoms and/or esophageal eosinophilia typically sets within 3 to 6 months^68^. During the maintenance phase half of initial dose of PPIs maintained clinical and histological remission in at least 75% of patients after 1 year^64^.

In long-term therapy, PPIs are also used as first-line treatment for EoE, given their ease of use, good cost-benefit ratio and no reported significant side effects to date as demonstrated in safety studies in adults who used PPIs at standard doses ranging from 5 to 12 years^65^.

Although preliminary studies show that P-CABs, particularly vonoprazan fumarate, may have similar efficacy to PPIs in EoE treatment, further studies are necessary before establishing their use in clinical practice^65^.

### What is the role of corticosteroids in the treatment of EoE?

#### Recommendation

Swallowed topical corticosteroids may be indicated as first-line therapy, for patients who fail to respond to PPIs or who present with more severe forms of the disease.

**Quality of evidence:** intermediate/high

**Delphi consensus level:** 100%

### Summary of evidence

Oral-administered topical corticosteroids, including PPIs, dietary therapy, and biologic agents, represent first-line treatment options for EoE^11^ ^,^ ^18^ ^,^ ^26^ ^,^ ^35^. Swallow topical corticosteroids may also be used in patients who have not responded to PPIs and diet, and as primary therapy in those with more aggressive disease^11^ ^,^ ^18^ ^,^ ^26^ ^,^ ^35^ ^,^ ^66^.

Corticosteroids primarily developed for inhalation such as fluticasone and budesonide are swallowed to exert local effects in the esophagus. In Brazil, these are off-label approaches, while in Europe and the United States, specific formulations of orodispersible tablets and oral suspensions of budesonide are available^57^ ^,^ ^67^ ^,^ ^68^.

A randomized clinical trial showed that orodispersible 1 mg budesonide tablets (two diary doses) led to histologic, endoscopic, and clinical improvement in 58% of patients at 6 weeks compared to 0% in the placebo group^69^. Sustained remission (48 weeks) was observed in 73.5% (0.5 mg twice daily) and 75% (1 mg twice daily) of patients compared to 4% in the placebo group^70^. The time for recurrence was 87 days mean in the placebo group versus 350 days mean in treated groups^70^.

The risk of oral candidiasis with topic-oral corticosteroids was about 5% highlighting the role of oral hygiene during treatment. A Cochrane review showed that topical corticosteroids reduce mucosal eosinophilia and stricture formation, achieving clinical, histological, and endoscopic remission, although not superior to PPIs^71^.

A meta-analyses confirmed the efficacy of swallowed topical corticosteroids versus placebo in EoE by inducing histological, clinical, and endoscopic responses, with good tolerability. Results showed complete histologic response (OR 35.82, 95%CI 14.98-85.64, *P*<0.0001; I2=0, *P*=0.72) e partial (OR 28.44, 95%CI 8.56-94.47, *P*<0.0001; I2=70%,=0.0009). Moreover, swallowed topical corticosteroids showed good clinical response (OR 2.53, 95%CI 1.14-5.60, *P*=0.02; I2=60%, *P*=0.02) and endoscopic responsiveness (OR 3.51, 95%CI 1.47-8.36, *P*=0.005; I2=0, *P*=0.57)^72^. It is worth noting that no topical formulation has been shown to be superior to any other in head-to-head studies^60^ ^,^ ^72^ ^,^ ^73^.

Other studies also comparing systemic to swallowed topical corticosteroids revealed similar efficacy in terms of clinical and histological response. However, the adverse events related to the use of systemic therapy preclude the recommendation of these drugs in the treatment of EoE^19^.

In clinical practice, therefore, swallowed topical corticosteroids can be used as first-line therapy, mainly in patients with long-lasting symptoms and esophageal signs of fibrostenosis as these patients are under high risk of obstruction, in cases of PPIs failure or those who declared preference for treatment with swallowed topical corticosteroids. 

### What is the role of immunobiologicals in the treatment of EoE?

#### Recommendation

Dupilumab is the first immunobiological approved by Anvisa (National Health Surveillance Agency) for the treatment of EoE with results that demonstrate efficacy in the treatment, both in the inflammatory phase and in the fibrostenotic phase of the disease.

**Quality of evidence:** intermediate/high

**Delphi consensus level:** 100%

### Summary of evidence

The goals of treatment in EoE include improvement of symptoms, remission of the esophageal eosinophilia and other histological abnormalities, improvement of endoscopic findings, enhancement of quality of life and esophageal function, minimization of adverse events related to treatment, prevention of disease progression, and subsequent complications^64^ ^,^ ^67^.

As EoE is an immune-mediated condition characterized by localized inflammatory and histological dysregulation besides inflammatory histopathological features, and progressive chronic dysfunction confined to the esophagus, biologic therapies are deemed as a very promising approach. 

Although the pathogenesis of EoE is not completely elucidated, pivotal features of the condition are well established to mention the Th2 inflammatory character. The Th2 path is characterized by increased production of interleukins (IL) IL-4, IL-5, and IL-13, which play a central role in the pathogenesis of EoE^11^ ^,^ ^74^. In knowledge these particularities many well established research fields are prone to explore possibilities which allow the development of molecules in a wide spectrum of targets to tackle the outcomes of altered inflammatory pathways. 

As IL-4 e IL-13 are involved in the Th2 inflammatory pathways and are key players in the pathogenesis of EoE, many targets in the IL-4 and IL-13 pathways have been considered strategic in the treatment of EoE^11^ ^,^ ^74^. Over the past decade, several biologic molecules were developed and initially appeared promising. On the other hand, while many have already been discarded, other molecules in varied phases of clinical trials were later found to produce unsatisfactory results and were discontinued such as mepolizumab, reslizumab, omalizumab, infliximab and lirentelimab^75^ ^-^ ^79^.

Conversely, dupilumab achieved efficacy and safety in phase 2 and 3 randomized clinical trials, leading to its approval to clinical use by the Food and Drug Administration (FDA) in USA in 2022^80^ ^,^ ^81^ and, in 2023, its subsequent approval by Anvisa81. Today, dupilumab is the unique monoclonal antibody approved to treat EoE.

Dupilumab is a monoclonal antibody that recognizes IL-4Rα and IL-13Rα1 receptors blocking the inflammatory signaling of IL-4 and IL-13^86^. It is also approved for the treatment of atopic dermatitis, asthma, and chronic rhinosinusitis with nasal polyps.

The results of a prospective, randomized, placebo-controlled study in three parts were observed:

Patients aged 12 years and older received subcutaneous dupilumab at a weekly dosage of 300 mg or placebo for 24 weeks. The dupilumab group presented higher histological response with less than 6 eosinophils/hpf compared to the placebo group (60% vs 5%; *P*<.001), and also presented a significant reduction in the dysphagia score (*P*<.001);

Another part of the sample was split in subgroups and received subcutaneous dupilumab at a weekly dosage of 300 mg, every two weeks or placebo for 24 weeks. The weekly dupilumab vs placebo dosing in part B replicated the results of part A (59% vs 6% for histologic response, *P*<.001; greater symptomatic improvement with dupilumab, *P*<.001). Dupilumab also improved the endoscopic severity score (EREFS) and histologic severity score (HSS) when compared with placebo in both A and B for diverse dose frequencies.

Patients completing Part A entered Part C, continuing weekly dupilumab 300 mg through week 52 (parts A-C group). Patients originally treated with placebo achieved similar results after switching to dupilumab^80^.

Real-world studies further support the use of dupilumab in EoE^82^. Meta-analyses demonstrated that despite generally positive results and high-quality studies, limitations included small sample size^82^. Subsequent meta-analyses confirmed these findings, showing reductions in esophageal eosinophilic infiltration and improvements in dysphagia symptoms^82^.

Dupilumab has been shown to be effective even in patients resistant to PPI and corticosteroid therapy and in those with more advanced fibrostenotic disease^82^. In special cases, dupilumab may be considered earlier, particularly in patients with allergic comorbidities associated to type 2 inflammatory diseases, such as asthma, atopic dermatitis, and chronic rhinosinusitis with nasal polyps^11^ ^,^ ^84^.

Other studies with approaches involving monoclonal antibodies like cendakimab (anti-IL-13), benralizumab (anti-IL-5), and antisialic acid-binding Ig-like lectin 8 (Siglec-8) (lirentelimab) are ongoing and, although not yet applicable to clinical practice, may contribute to a better understanding of interleukin pathway inhibition in achieving symptomatic and histological remission in EoE^75^ ^,^ ^79^ ^,^ ^83^ ^,^ ^84^.

### Is pharmacological maintenance treatment recommended for EoE?

#### Recommendation

Maintenance pharmacological treatment is recommended for all patients with EoE.

**Quality of evidence:** high

**Delphi agreement level:** 100%

### Summary of evidence

Due to the high risk of disease recrudescence when therapy is discontinued, it is suggested that maintenance treatment should be considered as long-term in patients with EoE who have achieved clinical and histological remission^11^. Additionally, evidence supports the importance of maintenance therapy because, although some patients do not develop strictures or food impactions, it is not possible to clearly identify which ones will develop complications. On the other hand, there is a limitation in published data, and prospective randomized studies defining optimal maintenance strategies are lacking. Furthermore, there are no studies for more than 12 months as maintenance treatment for EoE.

It is recommended that maintenance therapy initially be conducted with monotherapy, with drug selection personalized for each patient through a shared decision-making process between physician and patient^11^.

A prospective study involving 121 patients with EoE, 40 patients (33%) achieved complete remission with high doses of omeprazole. None of the patients achieving histological remission experienced symptom recurrence, although half of those with inflammatory recurrence maintained clinical remission. Following dose reduction to 40 mg of omeprazole once daily, 31/38 (81%) remained in complete remission. Among these, 15/18 (83%) were maintained on 20 mg of omeprazole once daily^85^. The conclusion was that most patients maintained sustained clinical and histological remission with a dose of omeprazole equal to or less than 40 mg^85^.

A study comprising 630 EoE patients treated with PPIs, the histological remission occurred in 48.8% and clinical remission in 71%. After achieving remission, dose reduction was effective in maintaining sustained remission in 69.9% of patients^11^. The success of maintenance therapy with PPIs appears closely related to the degree of response achieved during induction therapy^70^. The British Society of Gastroenterology (BSG) recommends considering long-term PPI maintenance therapy for patients in clinical and histological remission^11^.

Swallowed topical corticosteroids have shown very effective results. The benefit of orodispersible budesonide was observed in all groups of patients with EoE, with a mean time to relapse of 87 days in the placebo arm compared with >350 days in both arms of treatment with orodispersible budesonide^70^. This result surpassed that observed with previous studies using swallowed inhaled steroids like fluticasone or viscous budesonide paste^78^.

Despite limited evidence due to few controlled studies and the difficulty in defining effective maintenance strategies, the American Gastroenterological Association (AGA) recommends continuous use of swallowed topical corticosteroids for patients in remission following induction^57^. Similarly, the BSG recommends orodispersible budesonide for maintenance therapy in adults and adolescents^11^. Conversely, systemic corticosteroids are not recommended due to similar efficacy but greater adverse event profiles^86^.

Dellon et al.^80^ demonstrated in a study involving patients with EoE who were unresponsive to PPIs that maintenance treatment with dupilumab for 52 weeks was effective in maintaining clinical and histological remission in most patients.

### What are the complications of EoE?

#### Recommendation

Complications of EoE are not frequent, although when they occur, they generally require immediate medical intervention. Prolonged and uncontrolled esophageal inflammation can eventually lead to irreversible structural changes, resulting in tissue fibrosis, stenosis, and functional impairment of the organ.

**Quality of evidence:** intermediate

**Delphi level of agreement:** 92%

### Summary of evidence

Given the irreversible nature of complications and their important consequences with more invasive and higher-risk therapeutic interventions, it is essential to establish a prompt diagnosis of EoE and immediately implement the therapy.

The main complications of EoE result from the prolonged chronic inflammatory process, with subsequent morbidities from tissue fibrosis as the main consequence^87^.

### Esophageal impaction

The occurrence of partial esophageal obstruction due to the presence of rings or stenosis can lead to esophageal food impaction as a complication of EoE, requiring urgent removal through upper digestive endoscopy^53^ ^,^ ^87^ ^,^ ^88^.

### Esophageal perforation

Esophageal perforation is a potentially relevant complication of EoE, although few cases have been reported^88^. The presence of eosinophilic infiltrates constitutes a possible mechanism that causes epithelial inflammation to progress to a fibrostenotic condition. The progression of the condition can lead to dysphagia and food impaction, with the risk of esophageal perforation^89^ ^-^ ^91^.

### Esophageal dysmotility

The etiopathogenesis of esophageal dysmotility still unclear, but it may be associated with infiltration of the esophageal mucosa by eosinophils as the resulting interactions, such as fibroblasts with eosinophils, gastric eosinophilia and inflammatory cytokines^92^.

### Achalasia-like alterations

Achalasia rarely is associated with eosinophilic esophagitis, but motility changes similar to those that occur in achalasia esophageal eosinophilia may occur. This may result from the release of eosinophilic-secretory products, which peristalsis destabilization and relaxing the lower esophageal sphincter. The release of pro-fibrotic products also causes tissue remodeling, contributing to the motility issues^88^.

### Intramucosal esophagus dissection

This is an uncommon complication consisting of the dissection of deeper layers of the mucosa and/or submucosa, resulting in a false esophageal lumen^93^ ^,^ ^94^.

### Pulmonary aspiration

Pulmonary aspiration due to dysphagia with consequent pneumonia is a very rare complication that may eventually occur requiring hospitalization^87^ ^,^ ^95^.

### When is esophageal dilation recommended and what techniques are indicated?

#### Recommendation

Endoscopic dilation is effective in improving symptoms in patients with fibrostenotic disease. A luminal diameter of 16 mm is a satisfactory outcome.

**Quality of evidence:** high

**Delphi level of agreement:** 92%

### Summary of evidence

Endoscopic dilation is indicated for symptomatic esophageal strictures. It is an effective strategy for managing dysphagia resulting from strictures associated with EoE.

There are at least three groups of patients who may respond to dilation therapy:

Patients presenting dysphagia and strictures identified by upper gastrointestinal endoscopy;

Patients who present dysphagia in the absence of endoscopically apparent strictures. This is explained by the fact that strictures in EoE may extend longitudinally and, therefore, be underdiagnosed by upper gastrointestinal endoscopy, only evidenced on imaging tests;

Patients who do not complain about the presence of dysphagia due to adaptive eating behaviors and food avoidance, despite the presence of high-grade esophageal strictures (<10 mm)^96^.

A yet unanswered important question concerns the timing of esophageal dilation in EoE patients.

If a patient presents with a high-grade esophageal stricture, prolonged food impaction, or is unlikely to adhere to pharmacological therapy, dilation prior to medical therapy is acceptable. Conversely, dilation may be postponed in patients with mild to moderate strictures, with re-evaluation during follow-up endoscopy to assess the response to anti-inflammatory therapy^97^ ^-^ ^99^.

A meta-analysis of 27 studies involving 845 patients with EoE who underwent a total of 1,820 esophageal dilations demonstrated that dilation improved dysphagia in 95% of patients after the procedure (95%CI=90-98%). Complications included perforation, which occurred in 0.38% (95%CI: 0.18-0.85), hemorrhage, which occurred in 0.05% (95%CI: 0-0.3%), and hospitalization in 0.67% (95%CI: 0.3-1.1%)^100^.

Another systematic review and meta-analysis of 37 studies including 977 patients who underwent 2,034 dilations found a perforation rate of 0.033% (95%CI, 0-0.226%), bleeding in 0.028% (95%CI, 0-0.217%), and a hospitalization rate of 0.689% (95%CI, 0-1.42%)^101^.

Dilation does not impact the esophageal inflammatory process but provides symptom relief from dysphagia. Thus, ongoing esophageal inflammation and remodeling require repeated dilations^97^.

### Is follow-up recommended for patients with EoE?

#### Recommendation

Patients with EoE should be monitored with regular consultations and endoscopic examinations, even if asymptomatic.

**Quality of evidence:** intermediate

**Delphi level of agreement:** 100%

### Summary of evidence

Although recent data suggest that not all patients will progress to fibrostenotic disease and that different endotypes may exist, supporting an individualized approach, the vast majority of EoE patients will require maintenance therapy^102^.

There is, however, a lack of studies or recommendations regarding the best monitoring and follow-up strategy for these patients, as the correlation between symptoms and histological inflammation of the esophageal mucosa is only modest^103^.

Medium and long-term treatment will therefore depend on disease severity and response to therapy; periodic follow-up endoscopies may be recommended to monitor for complications or recurrences^29^.

**FIGURE 2:**
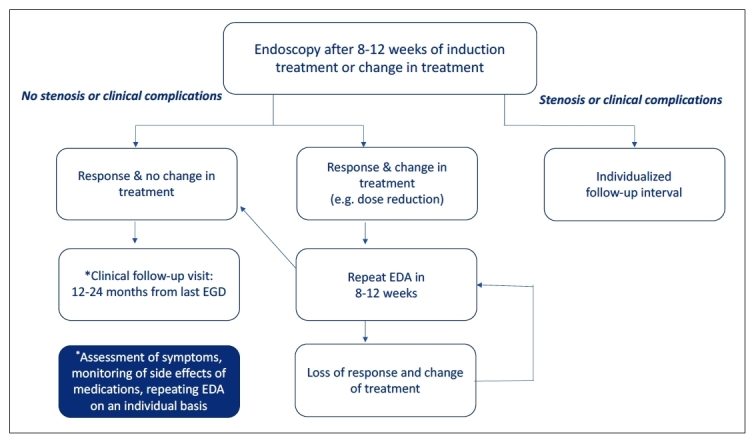
Follow-up algorithm for patients with EoE. Adapted from von Arnim et al.^104^.

Recently, an international panel of experts established recommendations and an algorithm (Figure 2) for monitoring these patients^104^. It is worth noting that current treatment may become ineffective over time and/or may have been discontinued by patients on their own initiative. Therefore, regular clinical follow-up is important and allows for the detection of these situations, as well as adjustments in management (dose and formulation), increasing adherence to therapy and preventing undesirable outcomes that compromise quality of life. In addition, it provides the possibility of new therapies being discussed in subsequent consultations^8^.

The decision on the periodicity of follow-up must consider factors such as disease clinical severity^105^, estimated risk of imminent food impaction, presence of strictures, and the mechanism of action and expected outcome of the intervention - whether pharmacological, dietary, or endoscopic - should be considered. Therefore, follow-up intervals of 6, 8, 10, 16, or even 24 months may be appropriate^8^.

The Clinical Severity Index (I-SEE) has recently been validated and may serve as a useful tool for individualizing patient management and monitoring intervals^105^.

In patients with significant initial esophageal diameter reduction, multiple dilations may be necessary. Initial esophageal diameter and histological remission are associated with successful outcomes in patients with severe strictures^106^.

### What is the importance of early diagnosis in EoE?

#### Recommendation

Early diagnosis and treatment reduce the development of EoE complications and improve quality of life.

**Quality of evidence:** intermediate/low

**Delphi agreement level:** 100%

### Summary of evidence

EoE is a chronic inflammatory esophageal disease that may progress from an inflammatory phenotype to a fibrostenotic phenotype if left untreated. Effective therapy can limit disease progression. The natural history of untreated EoE leads to subepithelial fibrosis, esophageal remodeling, and luminal strictures^43^.

In a study with 30 untreated adults followed for an average of 7.2 years, persistent dysphagia was observed in almost all patients, with subepithelial fibrosis detected in 86% of esophageal biopsies^107^.

A multicentric longitudinal study showed that up to 70% of untreated patients developed strictures^108^. The rate of fibrotic endoscopic findings may double (47% vs 88%) and the rate of strictures may increase nearly fourfold (17% vs 71%) when diagnosis occurs more than 20 years after symptom onset compared to diagnosis within 2 years^7^. In children, data suggest that subepithelial fibrosis and tissue remodeling may be reversible after treatment with topical steroids or dietary therapy^109^ ^,^ ^110^.

In advanced stages, esophageal damage has significant consequences for patients, including impaired quality of life, severe dysphagia, food impaction, and spontaneous esophageal perforation, emphasizing the critical importance of early diagnosis in preventing the development of EoE complications^111^.

### General approach to EoE treatment

Once EoE is diagnosed, both the inflammatory and fibrostenotic aspects of the disease must be addressed. All patients should receive anti-inflammatory treatment and be evaluated for fibrostenotic changes in the esophagus.

It is worth noting that EoE is a chronic disease, and any treatment adopted should be continued in the long term. There are three main options for anti-inflammatory therapy: pharmacologic treatment, dietary therapy, and biologic therapy.

As there is lack of comparative studies on safety and efficacy among treatment modalities until the present date, treatment options should be shared with the patient, considering important factors such as patient preference, easy of administration, accessibility, and cost.

Pharmacologic options include PPIs or swallowed topical corticosteroids. Although concurrent use may be considered in special cases, initial individualized use is preferred given the scarcity of data in the literature on combination therapy.

If an elimination diet is selected, empirical elimination diet is the most practical clinical approach, typically starting with elimination of one or two foods (cow’s milk and wheat), with broader restrictions applied as needed based on clinical response. Regardless of the initial treatment, treatment response should be evaluated via endoscopy with esophageal mucosal biopsies 8 to 12 weeks after therapy initiation.

In patients with a satisfactory response to treatment, this should be maintained and, in cases where patients opted for pharmacological treatment, a dose reduction may be attempted. However, monitoring of the treatment is essential, which includes, in addition to clinical evaluation, the mandatory performance of a new endoscopy with biopsies to assess the inflammatory response in the esophageal mucosa after reducing the dose of the medication.

If the response to the initial treatment is inadequate, there is the possibility of switching to another pharmacological option, empirical elimination diet or immunobiological dupilumab, again, according to the decision assumed together with the patient. For EoE patients with concomitant atopic diseases that are indications for dupilumab use, earlier start of dupilumab may be recommended in the treatment algorithm, as indicated by the physician.

In cases of stricture detected by endoscopy, dilation is recommended in order to achieve an esophageal diameter of 16 - 18 mm. This endoscopic approach should be considered in conjunction with ongoing anti-inflammatory therapy of the esophagus.

Dilation should also be considered for patients with persistent dysphagia despite adequate objective (endoscopic and histologic) treatment response.

## CONCLUSION

The importance of these FBG guidelines lies in its widespread use in order to support decision-making in clinical practice through adherence to proposed statements and recommendations. The suggested preferable approaches to patients with EoE were based on the growing evidence currently available, especially regarding new therapeutic options and follow-up issues. Still, there are unmet needs that will be addressed in the near future and development of guidelines will help to solve these problems. Multidisciplinary approach to EoE patients are of utmost importance to the extent that healthcare professionals, caregivers and patient´s family should be all involved in the process that will lead to the establishment of a standard of care to patients with EoE.

## References

[1] Hahn JW, Lee K, Shin JI (2023). Global incidence and prevalence of eosinophilic esophagitis, 1976-2022: a systematic review and meta-analysis. Clin Gastroenterol Hepatol.

[2] Bozorg SR, Söderling J, Mårild K (2024). Economic burden of eosinophilic esophagitis: a national wide cost-of-illness study. Am J Gastroenterol.

[3] Mukkada V, Falk GW, Eichinger CS (2018). Health-related quality of life and costs associated with eosinophilic esophagitis: a systematic review. Clin Gastroenterol Hepatol.

[4] Kim B, Rothenberg ME, Sun X (2024). Neuroimmune interplay during type 2 inflammation: symptoms, mechanisms, and therapeutic targets in atopic diseases. J Allergy Clin Immunol.

[5] Costa S, Aguiar JP, Oliveira MD (2025). Type 2 inflammation: a Portuguese consensus using Web-Delphi and decision conferencing (INFLAT2-PT). Expert Rev Clin Immunol.

[6] Lam AY, Lee JK, Coward S (2023). Epidemiologic burden and projections for eosinophilic esophagitis-associated emergency department visits in the United States: 2009-2030. Clin Gastroenterol Hepatol.

[7] Schoepfer AM, Safroneeva E, Bussmann C (2013). Delay in diagnosis of eosinophilic esophagitis increases risk for stricture formation in a time dependent manner. Gastroenterology.

[8] Chang NC, Thakkar KP, Ketchem CJ (2022). A gap in care leads to progression of fibrosis in eosinophilic esophagitis patients. Clin Gastroenterol Hepatol.

[9] Dellon ES, Muir AB, Katzka DA (2025). ACG Clinical Guideline: Diagnosis and management of eosinophilic esophagitis. Am J Gastroenterol.

[10] de Bortoli N, Visaggi P, Penagini R (2024). The 1st EoETALY consensus on the diagnosis and management of eosinophilic esophagitis: Definition, clinical presentation and diagnosis. Dig Liver Dis.

[11] Dhar A, Haboubi HN, Attwood SE (2022). British Society of Gastroenterology (BSG) and British Society of Pediatric Gastroenterology, Hepatology and Nutrition (BSPGHAN) joint consensus guidelines on the diagnosis and management of eosinophilic esophagitis in children and adults. Gut.

[12] Ntuli Y, Bough I Wilson M (2020). Recognizing eosinophilic esophagitis as a cause of food bolus obstruction. Frontline Gastroenterology.

[13] Murray FR, Kreienbuehl AS, Greuter T (2022). Diagnostic delay in patients with eosinophilic esophagitis has not changed since the first description 30 years ago: diagnostic delay in eosinophilic esophagitis. Am J Gastroenterol.

[14] Hawker S, Payne S, Kerr K (2002). Appraising the evidence: reviewing disparate data systematically. Qual Health Res.

[15] Dixon-Woods M & NHS Health Development Agency (2004). Integrative approaches to qualitative and quantitative evidence.

[16] Fairhurst KG, Huby G (1998). From trial data to practical knowledge: qualitative study of how general practitioners have accessed and used evidence about statin drugs in their management of hypercholesterolemia. BMJ.

[17] Dixon-Woods M, Cavers D, Agarwal S (2006). Conducting a critical Interpretive synthesis of the literature on access to healthcare by vulnerable groups. BMC Med Res Methodol.

[18] Dellon ES, Liacouras CA, Molina-Infante J (2018). Updated international consensus diagnostic criteria for eosinophilic esophagitis: proceedings of the AGREE Conference. Gastroenterology.

[19] Lucendo AJ, Molina-Infante J, Arias A (2017). Guidelines on eosinophilic esophagitis: evidence-based statements and recommendations for diagnosis and management in children and adults. United European Gastroenterol J.

[20] Loi R, Ceulemans M, Wauters L (2023). An update on eosinophilic esophagitis. Acta Gastro-Enterol Belgica.

[21] Dellon ES, Kim HP, Sperry SLE (2014). A phenotypic analysis shows that eosinophilic esophagitis is a progressive fibrostenotic disease. Gastrointest Endosc.

[22] Pentiuk S, Putnam PE, Collins MH (2009). Dissociation between symptoms and histologic severity in pediatric eosinophilic esophagitis. J Pediatr Nutr.

[23] Lucendo AJ, Arias-González L, Molina-Infante J (2017). Systematic review: health-related quality of life in children and adults with eosinophilic esophagitis-instruments for measurement and determinant factors. Aliment Pharmacol Ther.

[24] Furuta GT, Liacouras CA, Collins MH, Gupta SK (2007). Eosinophilic esophagitis in children and adults: a systematic review and consensus recommendations for diagnosis and treatment. Gastroenterology.

[25] Liacouras CA, Furuta GT, Hirano I (2011). Eosinophilic esophagitis: updated consensus recommendations for children and adults. J Allergy Clin Immunol.

[26] Dellon ES, Gonsalves N, Hirano I (2013). ACG clinical guideline: evidenced based approach to the diagnosis and management of esophageal eosinophilia and eosinophilic esophagitis (EEo). Am J Gastroenterol.

[27] Dellon ES, Gibbs WB, Fritchie KJ (2009). Clinical, endoscopic and histologic findings distinguish eosinophilic esophagitis from gastroesophageal reflux disease. Clin Gastroenterol Hepatol.

[28] Schoepfer AM, Simko A, Bussmann C. (2018). Eosinophilic esophagitis: relationship of subepithelial eosinophilic inflammation with epithelial histology, endoscopy, blood eosinophils, and symptoms. Am J Gastroenterol.

[29] Hiremath GS, Hameed F, Pacheco A (2015). Esophageal food impaction and eosinophilic esophagitis: a retrospective study, systematic review and meta-analysis. Dig Dis Sci.

[30] Chang JW, Olson S, Kim JY (2019). Loss to follow-up after food impactation among patients with and without eosinophilic esophagitis. Dis Esophagus.

[31] Gómez-Aldana A, Jaramillo-Santos M, Delgado A (2019). Eosinophilic esophagitis: Currents concepts in diagnosis and treatment. World J Gastroenterol.

[32] Shaheen NJ, Mukkada V, Eichinger CS (2018). Natural history of eosinophilic esophagitis: a systematic review of epidemiology and disease course. Dis Esophagus.

[33] Leigh LY, Spergel JM (2019). An in-depth characterization of a large cohort of adult patients with eosinophilic esophagitis. Ann Allergy Asthma Immunol.

[34] Torrijos G Sanches M, Donado P. (2017). Eosinophilic esophagitis: demographic, clinical, endoscopic, histologic, and topic characteristics od children and teenager I a region in Central Spain. J Investig Allergol Clin Immunol.

[35] Aceves SS, Alexander JA, Baron TH (2022). Endoscopic approach to eosinophilic esophagitis: American Society for Gastrointestinal Endoscopy Consensus Conference. Gastrointest Endosc.

[36] Hirano I, Moy N, Heckman MG (2013). Endoscopic assessment of the esophageal features of eosinophilic esophagitis: validation of a novel classification and grading system. Gut.

[37] Kim HP, Vance RB, Shaheen NJ (2012). The prevalence and diagnostic utility of endoscopic features of eosinophilic esophagitis: A meta-analysis. Clin Gastroenterol Hepatol.

[38] Dellon ES, Cotton C, Gebhart J (2016). Accuracy of eosinophilic esophagitis endoscopic reference score in diagnosis and determining response to treatment. Cli Gastroenterol Hepatol.

[39] Collins MH (2014). Histopathologic features of eosinophilic esophagitis and eosinophilic gastrointestinal diseases. Gastroenterol Clin North Am.

[40] Dellon ES, Speck O, Woodward K. (2015). Distribution and variability of esophageal eosinophilia in patients undergoing upper endoscopy. Mod Pathol.

[41] Dellon ES (2024). Eosinophilic esophagitis: what’s in a name?. Dig Dis Sci.

[42] Collins MH, Martin LJ, Wen T (2020). Eosinophilic esophagitis histology remission score: signiﬁcant relations to measures of disease activity and symptoms. J Pediatr Gastroenterol Nutr.

[43] Bortoli De (2024). The 1st EoETALY Consensus on the diagnosis and management of eosinophilic esophagitis - definition, clinical presentations and diagnosis. Dig Liver Dis.

[44] Salek J, Clayton F, Vinson L (2015). Endoscopic appearance and location dictate diagnostic yield of biopsies in eosinophilic esophagitis. Aliment Pharmacol Ther.

[45] Okimoto E, Ishimura N, Okada M. (2017). Specific locations of linear furrows in patients with esophageal eosinophilia. Dig Endosc.

[46] Hashimoto A, Uemura R, Sawada A. (2019). Optimal biopsy protocol to evaluate histological effectiveness of proton pump inhibitor therapy in patients with eosinophilic esophagitis. Digestion.

[47] Pitsios C, Vassilopoulou E, Pantavou K (2022). Allergy-test-based elimination diets for the treatment of eosinophilic esophagitis: a systematic review of their efficacy. J Clin Med.

[48] Capucilli P, Hill DA (2019). Allergic comorbidity in eosinophilic esophagitis: mechanistic relevance and clinical implications. Clin Rev Allergy Immunol.

[49] Lim AH, Wong S, Nguyen NQ (2021). Eosinophilic esophagitis and IgG4: Is there a relationship?. Dig Dis Sci.

[50] Chang JW, Kliewer K, Haller E, Lynett A (2023). Consortium of eosinophilic gastrointestinal disease researchers. Development of a practical guide to implement and monitor diet therapy for eosinophilic esophagitis. Clin Gastroenterol Hepatol.

[51] Philpott H, Nandurkar S, Royce SG (2016). Allergy tests do not predict food triggers in adult patients with eosinophilic esophagitis. A comprehensive prospective study using five modalities. Aliment Pharmacol Ther.

[52] Eckmann JD, Ravi K, Katzka DA (2018). Efficacy of atopy patch testing in directed dietary therapy of eosinophilic esophagitis: a pilot study. Dig Dis Sci.

[53] Arias A, González-Cervera J, Tenias JM (2014). Efficacy of dietary interventions for inducing histologic remission in patients with eosinophilic esophagitis: a systematic review and meta-analysis. Gastroenterology.

[54] Quigley EMM (2024). Can diet change the natural history of gastrointestinal diseases?. JGH Open.

[55] Muir A, Falk GW (2021). Eosinophilic esophagitis: a review. JAMA.

[56] Lucendo AJ (2022). Nutritional approach to eosinophilic esophagitis: which diet and when. Minerva Gastroenterology.

[57] Hirano I, Chan ES, Ma Rank (2020). AGA Institute and the Joint Task Force on Allergy-immunology practice parameters clinical guidelines for the management of eosinophilic esophagitis. Gastroenterology.

[58] Gonsalves N, Yang GY, Doerfler B (2012). Elimination of diet effectively treats eosinophilic esophagitis in adults: food reintroduction identifies causative factors. Gastroenterology.

[59] Wang R, Hirano I, Doerfler B (2018). Assessing adherence and barriers to long term elimination diet therapy in adults with eosinophilic esophagitis. Dig Dis Sci.

[60] Ma Rank, Sharaf RN, Furuta GT (2020). Technical review on the management of eosinophilic esophagitis : a report from the AGA Institute and the Joint task-force on Allergy-Immunology Practice Parameters. Gastroenterology.

[61] Molina-Infante J, Rivas MD, Hernandez-Alonso M (2014). Proton pump inhibitor-responsive esophageal eosinophilia correlates with downregulation of eotaxin-3 and Th2 cytokines overexpression. Aliment Pharmacol Ther.

[62] Lucendo AJ, Arias A, Molina-Infante J (2016). Efficacy of proton pump inhibitor drugs for inducing clinical and histologic remission in patients with symptomatic esophageal eosinophilia: a systematic review and meta-analysis. Clin Gastroenterol Hepatol.

[63] Miehlke S, Von Arnim U, Schlag C (2019). Clinical management of eosinophilic esophagitis - a nationwide survey among gastroenterologists in Germany. Z Gastroenterol.

[64] Franciosi JP, Mougey EB, Dellon ES (2022). Proton pump inhibitor therapy for eosinophilic esophagitis: history, mechanisms, efficacy, and future Directions. J Asthma Allergy.

[65] DeLay K, Tappata M, Huang KZ (2020). Long-term continued proton pump inhibitor use is common in patients diagnosed with eosinophilic esophagitis despite failure of histologic response: data from a two-centre study: Long-term PPI Use in Patients with EoE. GastroHep.

[66] Gonsalves NP, Aceves SS (2020). Diagnosis and treatment of eosinophilic esophagitis. J Allergy Clin Immunol.

[67] Hirano I, Furuta GT (2020). Approaches and challenges to management of pediatric and adult patients with eosinophilic esophagitis. Gastroenterology.

[68] Reddy A, Ashat D, Murali AR (2020). Recent insights on the use of topical steroids in eosinophilic esophagitis. Expert Rev Gastroenterol Hepatol.

[69] Lucendo AJ, Miehlke S, Schlag C (2019). International EOS-1 Study Group. Efficacy of budesonide orodispersible tablets as induction therapy for eosinophilic esophagitis in a randomized placebo-controlled trial. Gastroenterology.

[70] Straumann A, Lucendo AJ, Miehlke S (2020). Budesonide orodispersible tablets maintain remission in a randomized, placebo-controlled trial of patients with eosinophilic esophagitis. Gastroenterology.

[71] Gupta M, Grinman M (2024). Diagnosis and management of eosinophilic esophagitis. CMAJ.

[72] Lipka S, Kumar A, Miladinovic B (2016). Systematic review with network meta-analysis: comparative effectiveness of topical steroids vs. PPIs for the treatment of the spectrum of eosinophilic esophagitis. Aliment Pharmacol Ther.

[73] Dellon ES, Woosley JT, Arrington A (2019). Efficacy of budesonide vs fluticasone for initial treatment of eosinophilic esophagitis in a randomized controlled trial. Gastroenterology.

[74] Dellon ES, Spergel JM (2023). Biologics in eosinophilic gastrointestinal diseases. Ann Allergy Asthma Immunol.

[75] Assa’ad AH, Gupta SK, Collins MH (2011). An antibody against IL-5 reduces numbers of esophageal intraepithelial eosinophils in children with eosinophilic esophagitis. Gastroenterology.

[76] Markowitz JE, Jobe L, Miller M (2018). Safety and efficacy of reslizumab for children and adolescents with eosinophilic esophagitis treated for 9 years. J Pediatr Gastroenterol Nutr.

[77] Dellon ES, Peterson KA, Murray JA (2020). Anti-Siglec-8 antibody for eosinophilic gastritis and duodenitis. N Engl J Med.

[78] Straumann A, Bussmann C, Conus S (2008). Anti-TNF-alpha (infliximab) therapy for severe adult eosinophilic esophagitis. J Allergy Clin Immunol.

[79] Foroughi S, Foster B, Kim N (2007). Anti-IgE treatment of eosinophil-associated gastrointestinal disorders. J Allergy Clin Immunol.

[80] Dellon ES, Rothenberg ME, Collins MH (2022). Dupilumab in adults and adolescents with eosinophilic esophagitis. N Eng J Med.

[81] Harb H, Chatila TA (2020). Mechanisms of dupilumab. Clin Exp Allergy.

[82] Spergel BL, Ruffner MA, Godwin BC (2022). Improvement in eosinophilic esophagitis when using dupilumab for other indications or compassionate use. Ann Allergy Asthma Immunol.

[83] Gómez ARG, Sotomayor JVM, Romo JBJR (2022). Symptoms and complications that require urgent treatment and upper digestive comorbidities in eosinophilic esophagitis. Dig Liver Dis.

[84] Hirano I, Collins MH, Assouline-Dayan Y (2019). RPC4046, a Monoclonal antibody against IL13, reduces histologic and endoscopic activity in patients with eosinophilic esophagitis. Gastroenterology.

[85] Gómez-Torrijos E, García-Rodríguez R, Castro-Jiménez A (2016). The efficacy of step-down therapy in adult patients with proton pump inhibitor-responsive esophageal eosinophilia. Aliment Pharmacol Ther.

[86] De Bortoli N, Visaggi P, Penagini R (2024). The 1st EoETALY Consensus on the diagnosis and management of eosinophilic esophagitis - current treatment and monitoring. Dig Liver Dis.

[87] De Bortoli N, Penagini R, Savarino E, Marchi S (2017). Eosinophilic esophagitis: Update in diagnosis and management. Position paper by the Italian Society of Gastroenterology and Gastrointestinal Endoscopy (SIGE). Digestive and Liver Disease.

[88] Khan S, Guo X, Iqbal M (2021). An update on eosinophilic esophagitis: etiological factors, coexisting diseases, and complications. Digestion.

[89] Issa D, Alwatari Y, Smallfield GB (2019). Spontaneous transmural perforation in eosinophilic esophagitis: RARE case presentation and role of esophageal stenting. J Surg Case Rep.

[90] Lucendo AJ, Friginal-Ruiz AB, Rodriguez B (2011). Boerhaave’s syndrome as the primary manifestation of adult eosinophilic esophagitis. Two case reports and a review of the literature. Dis Esophagus.

[91] Vernon N, Mohananey D, Ghetmiri S (2014). Esophageal rupture as a primary manifestation in eosinophilic esophagitis. Case Rep Med.

[92] Nurko S, Rosen R (2008). Esophageal dysmotility in patients who have eosinophilic esophagitis. Gastrointest Endosc Clin N Am.

[93] Fianchi F, De Matteis G, Cianci R (2019). Acute intramucosal dissection in eosinophilic esophagitis. Clin J Gastroenterol.

[94] Sgro A, Betalli P, Battaglia G (2012). An unusual complication of eosinophilic esophagitis in an adolescent: intramural esophageal dissection. Endoscopy.

[95] Biedermann I, Straumann A, Greuter T (2021). Eosinophilic esophagitis -established facts, and new horizons. Semin Immunopathol.

[96] Hirano I (2018). How to approach a patient with eosinophilic esophagitis. Gastroenterology.

[97] Runge TM, Eluri S, Cotton CC (2016). Outcomes of esophageal dilation in eosinophilic esophagitis: safety, efficacy, and persistence of the fibrostenotic phenotype. Am J Gastroenterol.

[98] Richter JE (2016). Eosinophilic esophagitis dilation in the community - try it - you will like it-but start low and go slow. Am J Gastroenterol.

[99] Carlson DA, Hirano I, Zalewski A (2017). Improvement in esophageal distensibility in response to medical and diet therapy in eosinophilic esophagitis. Clin Transl Gastroenterol.

[100] Moawad FJ (2017). Systematic review with meta-analysis: Endoscopic dilation is highly effective and safe in children and adults with eosinophilic esophagitis. Aliment. Pharmacol Ther.

[101] Dougherty M, Runge TM, Eluri S, Dellon ES (2017). Esophageal dilation with either bougie or balloon technique as a treatment for eosinophilic esophagitis: A systematic review and meta-analysis. Gastrointest. Endosc.

[102] Shoda T, Wen T, Aceves SS (2018). Consortium of Eosinophilic Gastrointestinal Disease Researchers (CEGIR). Eosinophilic esophagitis endotype classification by molecular, clinical, and histopathological analyses: a cross-sectional study. Lancet Gastroenterol Hepatol.

[103] Safroneeva E, Straumann A, Coslovsky M (2016). International Eosinophilic Esophagitis Activity Index Study Group. Symptoms have modest accuracy in detecting endoscopic and histologic remission in adults with eosinophilic esophagitis. Gastroenterology.

[104] Von Arnim U, Bierdemann L, Aceves SS (2023). Monitoring patients with eosinophilic esophagitis in routine clinical practice - International Expert Recommendations. Clin Gastroenterol Hepatol.

[105] Dellon ES, Khoury P, Muir AB (2022). A clinical severity index for eosinophilic esophagitis: development, consensus, and future directions. J Allergy Clin Immunol.

[106] Kim JP, Weingart G, Hiramoto B (2020). Clinical outcomes of adults with eosinophilic esophagitis with severe stricture. Gastrointest Endosc.

[107] Straumann A (2003). Natural history of primary eosinophilic esophagitis: a follow-up of 30 adult patients for up to 11.5 years. Gastroenterology.

[108] Singla MB (2015). Early comparison of inflammatory vs. fibrostenotic phenotype in eosinophilic esophagitis in a multicenter longitudinal study. Clin Transl Gastroenterol.

[109] Lieberman JA, Morotti RA, Konstantinou GN (2012). Dietary therapy can reverse esophageal subepithelial fibrosis in patients with eosinophilic esophagitis: a historical cohort. Allergy.

[110] Rajan J, Newbury RO, Anilkumar A (2016). Long-term assessment of esophageal remodeling in patients with pediatric eosinophilic esophagitis treated with topical corticosteroids. J Allergy Clin Immunol.

[111] Dellon ES, Hirano I (2018). Epidemiology and Natural History of Eosinophilic Esophagitis. Gastroenterology.

